# Assessing Perioperative Anesthesia Safety in Urological Surgeries: A Comprehensive Study of Critical Incidents

**DOI:** 10.7759/cureus.77361

**Published:** 2025-01-13

**Authors:** Pallavi Waghalkar, Vishal Pandit, Sunil Chapane, Ishita Lanjewar, Kshitij Sonawale, Alhad A Mulkalwar

**Affiliations:** 1 Anesthesiology, Seth Gordhandas Sunderdas (GS) Medical College and King Edward Memorial (KEM) Hospital, Mumbai, IND; 2 Anesthesiology, Employees' State Insurance (ESI) Mahatma Gandhi Memorial Hospital, Mumbai, IND; 3 Medicine, Seth Gordhandas Sunderdas (GS) Medical College and King Edward Memorial (KEM) Hospital, Mumbai, IND; 4 Pharmacology, Dr. D. Y. Patil Medical College, Hospital and Research Centre, Dr. D. Y. Patil Vidyapeeth (Deemed to be University), Pune, IND

**Keywords:** anesthesia, asa grade, clinical observations, critical incidents, lithotomy position, procedural vigilance

## Abstract

Background

Anesthesia-related critical incidents are significant causes of preventable harm during surgeries, particularly in specialized fields such as urology as they pose unique challenges, including advanced patient age, comorbidities, and complex procedures, which heighten anesthesia-related risks. These incidents are influenced by human errors, patient factors, and procedural complexity. Despite global advancements in safety protocols, there remains a need for standardized reporting and analysis of such incidents, especially in low/middle-income countries (LMICs).

Methods

A prospective and retrospective observational study was conducted from 2019 to 2022. Data was collected from the anesthesia database and perioperative records of 2,541 patients who underwent urological surgeries. Two hundred forty-one (9.48%) patients experienced critical incidents. Data included patient demographics such as age and gender, comorbidities, surgery details, and the timing and recognition of incidents. Incidents were divided into cardiovascular, respiratory, airway, central nervous system (CNS), and miscellaneous categories. A root cause analysis identified human and systemic factors.

Results

The incidence rate of critical incidents was 9.83%. The most common age group was 31-40 years (20.75%), and 71.36% of patients were men. Most incidents occurred in American Society of Anesthesiologists (ASA) Grade I patients (58.09%) and during the induction phase of anesthesia (41.49%). Cardiovascular incidents were the most frequent (23.24%), followed by airway issues (12.86%). Human factors, such as the lack of skill (43.98%), vigilance (22.41%), and judgment (16.18%), were the leading causes. Surgical position, especially lithotomy, was significantly associated with incidents (p<0.0001), and a significant association between the time to recognition of an incident and the mode of recognition of the incident was also found (p<0.0001). Notably, 98.75% of incidents were deemed preventable with existing protocols.

Conclusion

Human factors, especially the lack of skill, vigilance, and judgment, emerged as the primary contributors to critical incidents in anesthetic procedures. Emphasizing improved training, vigilance during induction, and attention to patient positioning can enhance patient safety. Most incidents were preventable, highlighting the need for better reporting and preventive protocols.

## Introduction

Anesthesia-related critical incidents refer to preventable events associated with anesthesia administration that result in undesirable patient outcomes. In specialized surgical fields such as urology, patient safety and outcome quality face significant challenges, owing to patient factors and the nature of the procedure. The branch of uro-anesthesia involves managing a demographically diverse patient population with underlying comorbidities, complicating anesthesia administration. Literature suggests a need to better understand and mitigate factors contributing to anesthesia-related complications in urological surgeries, particularly in resource-constrained settings where the adoption of safety protocols is inconsistent [1]. A review conducted in 2020 on anesthetic considerations in urological surgeries highlighted the importance of tailored anesthesia for elderly patients undergoing minimally invasive urological surgeries, covering critical factors such as age, sex, comorbidities, and anticipated blood loss [1]. This aligns with another prospective study conducted in India on critical incidence reporting, which found that children between zero and 10 years were most frequently affected by critical incidents, underscoring the role of patient demographics in incident occurrence and emphasizing the need for individualized anesthesia protocols [2].

The role of this study is to sound another perspective on the controversies that exist among the existing literature. For instance, Zeng et al.'s study illustrates the relationship between the American Society of Anesthesiologists (ASA) status and perioperative risk, showing that patients with higher ASA grades are at greater risk [3]. Specifically, ASA 5 patients had 13.7 times the odds of experiencing incidents compared to ASA 1 patients. This was in contrast to another study that reported a greater risk was posed to ASA 1 patients, a lower incidence (0.79%) but higher mortality (28.57%) [2,3]. Discrepancies about the stage of surgery most prone to critical incidents, causes that predispose to critical incidents, and patient factors also exist without an accepted consensus. Furthermore, research shows that many preventable incidents often go unreported [4]. Recent policy documents, such as the National Accreditation Board for Hospitals (NABH) 5th edition of Standards (April 2020), emphasize the importance of incident reporting as a crucial step in addressing these injuries [5]. According to the World Federation of Societies of Anesthesiologists (WFSA), the reporting and analysis of critical incidents are essential for continuously improving clinical practices and reducing anesthesia-related adverse outcomes. Still, there remains a significant gap in the availability and consistency of data on critical incident reporting, particularly in low/middle-income countries (LMICs), where various obstacles hinder effective implementation [6].

This study aimed to analyze perioperative critical incidents, specifically in the context of uro-anesthesia within uro-surgery operation theatres. This included examining contributing factors to identify preventable elements and areas for improvement. The investigators also wished to determine the incidence of critical incidents occurring in the uro-surgery operation theatre and to assess their association with preoperative ASA status. Additionally, the study aimed to investigate the relationship between critical incidents and various factors related to surgery, anesthesia, and patient characteristics. Furthermore, the study sought to explore the occurrence of critical incidents concerning the specific stage of the procedure in which they occurred and to evaluate the association between critical incidents and the positioning of the patient during surgery. This would lay the groundwork for targeted interventions to enhance perioperative safety in this high-risk field.

## Materials and methods

This prospective observational study spanned over two years. It was conducted using the records of patients receiving anesthesia in the uro-surgery operating theatre (OT) of King Edward Memorial Hospital, a tertiary care hospital in Western India. This study utilized a secondary database collection method. Data was derived from the department of anesthesia's database and perioperative records. It was validated through critical review. This study was conducted following approval from the institutional ethics committee of King Edward Memorial Hospital (approval number: EC/OA-58/2019). Written informed consent was obtained from every patient enrolled in the prospective phase during the preoperative assessment. For the retrospective analysis of the 2019 cases, a waiver of consent was granted. The study adhered to ethical guidelines for patient safety and confidentiality throughout its duration.

The study commenced in 2019; however, due to the COVID-19 pandemic, it was extended to 2022 for prospective data collection and the retrospective analysis of the 2019 data. In the study, records of all the 2,541 patients in the hospital who received anesthesia for urological surgeries were screened. Two hundred forty-one (9.48%) patients were found to have experienced critical incidents during this observational period and were included in the study. The inclusion criteria for the study consisted of all patients receiving anesthesia in the uro-surgery operation theatre (OT) over a two-year period. Patients who underwent urological surgeries, namely, nephrectomy, cystectomy, transurethral resection of the prostate (TURP), nephrolithotomy, and ureteroscopy, were chosen. The patients who refused to provide consent, pregnant women, and those with impaired decision-making capacity were excluded. The participants were recruited on the day of surgery during the preoperative assessment. In the prospective phase, patients were monitored intraoperatively and postoperatively for two hours in the postanesthesia high dependency unit (PAHDU) or ward. For the retrospective phase, patient records from 2019 were analyzed to identify significant critical incidents. Standard ASA monitoring, as per OT protocol, included ECG, pulse rate, pulse oximetry, end-tidal CO₂, blood pressure, temperature, and urine output, with invasive monitoring as needed. Critical incidents were documented by trained anesthetists in the department of anesthesia's database and managed according to the standard operating protocol. A case record form was used to classify these incidents, and a root cause analysis was performed by co-investigators to identify contributing factors. No corrective measures were part of this study. Data was analyzed using SPSS software version 29.0.2.0 (IBM Corp., Armonk, NY) [7].

Critical incidents in the perioperative setting were classified into five categories: cardiovascular, respiratory, airway, central nervous system (CNS), and miscellaneous. Data collected included diagnosis, patient demographics, details of the surgery, the location and timing of incidents, recognition time, and the potential for anticipation and prevention. The analysis of causative factors focused on human elements such as skill, judgment, and communication, along with equipment, surgical, patient, organizational, and pharmacological factors that led to these incidents.

## Results

The mean age of the patients was 44.29±18.05 years, ranging from 1.5 to 84 years. The largest age group was 31-40 years (20.75%, 50), followed by 41-50 years (18.67%, 45) as shown in Table 1. The majority of the patients were men (71.36%, 172), while 28.63% (69) were women.

**Table 1:** Distribution of Study Subjects by Age The data has been represented as numbers (N) and percentage (%)

Age Group (Years)	Number (N)	Percentage (%)
0-10	5	2.07
11-20	15	6.22
21-30	39	16.18
31-40	50	20.75
41-50	45	18.67
51-60	37	15.35
61-70	31	12.86
71-80	18	7.47
>80	1	0.41
Total	241	100

Most patients were classified under ASA Grade I (58.09%, 140), followed by Grade II (34.02%, 82) and Grade III (7.88%, 19) as highlighted in Table 2.

**Table 2:** Distribution of Study Subjects by ASA Grade The data has been represented as numbers (N) and percentage (%) ASA: American Society of Anesthesiologists

ASA Grade	Number (N)	Percentage (%)
Grade I	140	58.09
Grade II	82	34.02
Grade III	19	7.88
Total	241	100

Among the procedures performed (Table 3), percutaneous nephrolithotomy (PCNL) was the most common (28.63%, 69), followed by laparoscopic nephrectomy (10.79%, 26) and transurethral resection of the prostate (TURP) (9.96%, 24). Other surgeries included were cystoscopy (6.22%, 15), ureteroscopic lithotripsy (5.81%, 14), urethroplasty (5.39%, 13), laparoscopic pyeloplasty (3.73%, nine), ureter reimplant (1.66%, four), transurethral resection of bladder tumor (1.66%, four), cystolithotripsy (1.24%, three), laparoscopic ureterostomy (1.24%, three), ureteroscopy (1.24%, three), vesicovaginal fistula repair (1.24%, three), visual internal urethrotomy (1.24%, three), open bladder diverticular repair (0.83%, two), total penectomy (0.83%, two), clot evacuation (0.41%, one), high orchiectomy (0.41%, one), radical cystectomy with ileal conduit (0.41%, one), renal cell CA (0.41%, one), laparoscopic radical cystectomy with ileal conduit (0.41%, one), ileal ureter (0.41%, one), retrograde intrarenal surgery (0.41%, one), redo hypospadias (0.41%, one), and duodenojejunal (DJ) stent removal (0.41%, one). These surgeries included 127 cases of subarachnoid block (regional anesthesia), 22 cases of subarachnoid blocks converted to general anesthesia, and 88 cases of general anesthesia.

**Table 3:** Distribution of Study Subjects by Surgical Procedure The data has been represented as numbers (N) and percentage (%) PCNL, percutaneous nephrolithotomy; TURP, transurethral resection of the prostate; URSL, ureteroscopic lithotripsy

Surgery Planned	Number of Patients (N)	Percentage (%)
PCNL	69	28.63
Laparoscopic Nephrectomy	26	10.79
TURP	24	9.96
Cystoscopy	15	6.22
URSL	14	5.81

**Figure 1:**
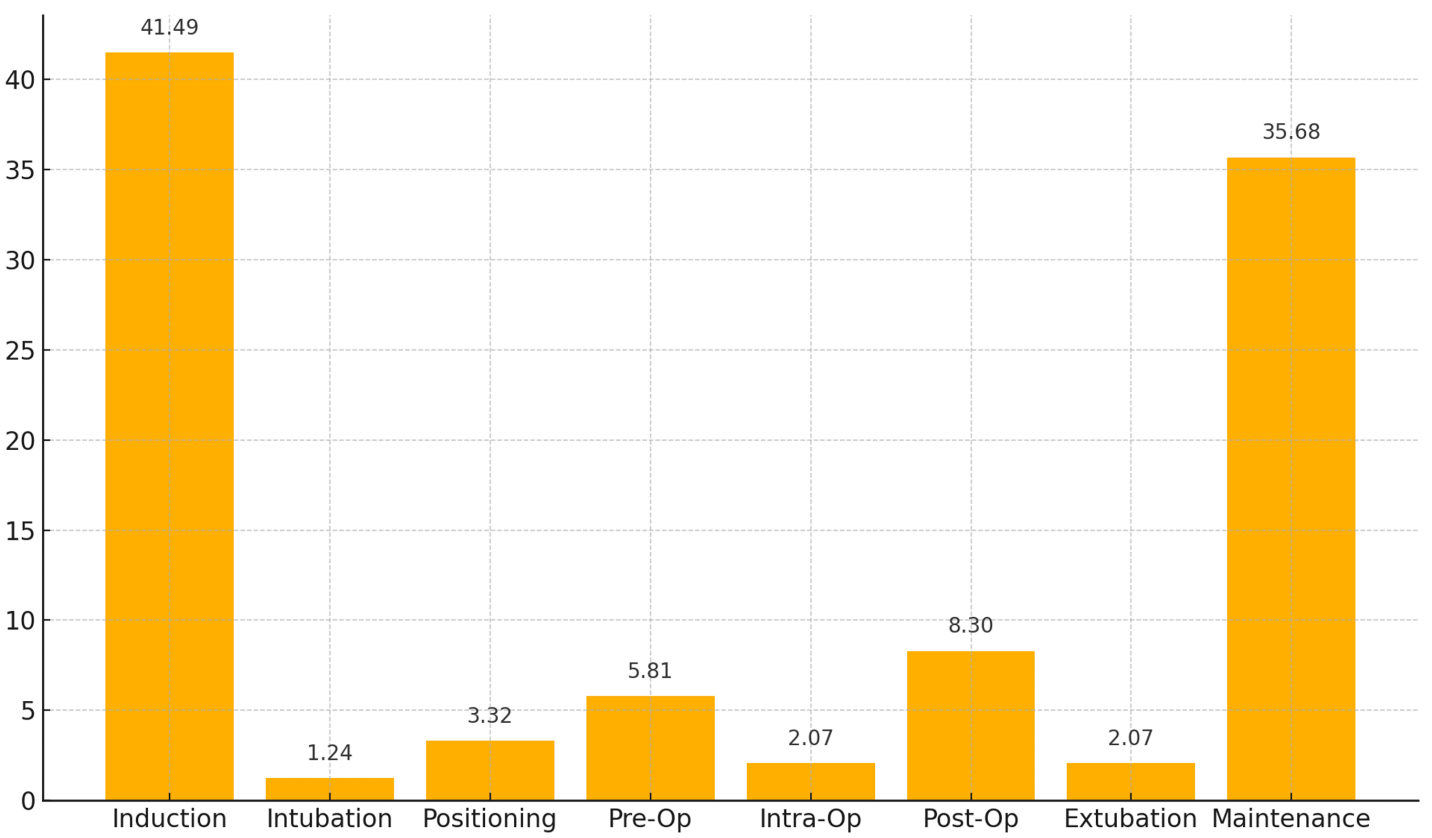
Distribution of Study Subjects by Timing Y-axis: the data has been represented as percentage (%). X-axis: timings of critical incidents Op: operation

Mainly, critical incidents occurred during the induction phase (41.49%, 100), followed by the maintenance phase (35.68%, 86). Critical incidents during intubation, positioning, extubation, and postoperative periods were less frequent (Figure 1).

In 54.36% (131) of cases, the critical incident was recognized within five minutes, while in 36.51% (88), recognition occurred within 5-10 minutes. The primary mode of recognition was clinical observation (74.68%, 180), while a combination of clinical observation and monitoring was used in 23.65% (57) of cases. The chi-square test revealed a significant association between time to recognition and the mode of recognition of incidents (chi-square=137.12; p<0.0001), indicating that the method used to recognize incidents is statistically related to the time it takes for recognition.

The most frequent critical incidents were cardiovascular incidents at 23.24% (56) and airway-related issues at 12.86% (31). Central nervous system (CNS) incidents accounted for 2.07% (five) of the cases. The remaining incidents were miscellaneous and could not be classified under one category (Table 4).

**Table 4:** Distribution of the Type of Critical Incidents The data has been represented as numbers (N) and percentage (%) CNS, central nervous system; CVS, cardiovascular system

Type of Critical Incident	Number (N)	Percentage (%)
Cardiovascular	56	23.24
Airway	31	12.86
CNS	5	2.07
CVS+Airway	3	1.24

**Figure 2:**
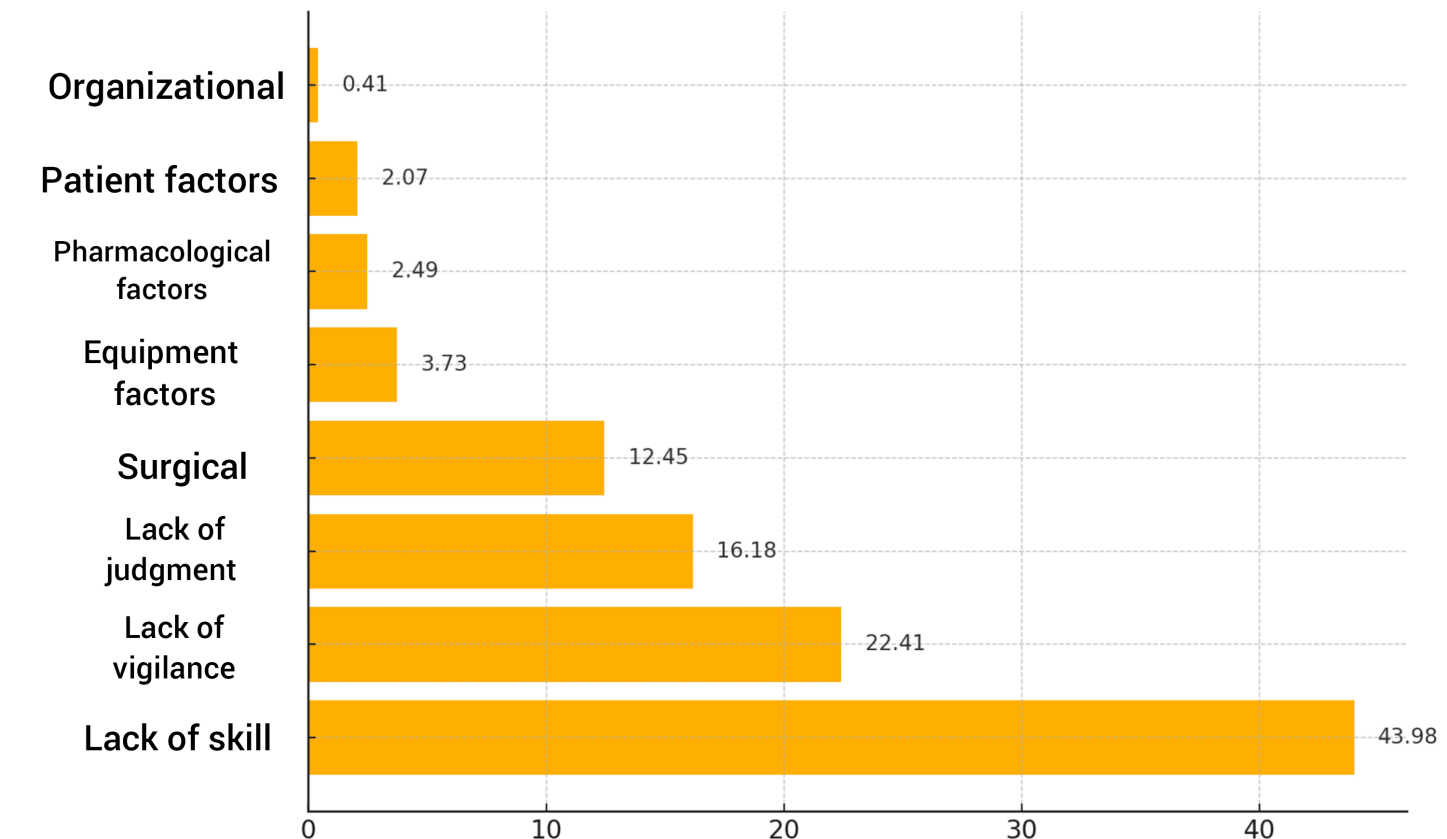
Causative Factors Associated With Critical Incidents Y-axis: causative factors of critical incidents. X-axis: percentage (%)

As depicted in Figure 2, human factors dominated as the primary cause of critical incidents, with the lack of skill (43.98%, 106), the lack of vigilance (22.41%, 54), and the lack of judgment (16.18%, 39) being the most frequently cited reasons. Surgical factors contributed to 12.45% (30) of the incidents, while equipment and pharmacological factors were less common (3.73%, nine, and 2.49%, six, respectively).

Spearman correlation analysis revealed a weak positive correlation (r=0.027) between the surgical position and ASA grade, suggesting minimal association between these factors. However, a chi-square analysis revealed a significant association between lithotomy position and causative factors (p<0.0001) (chi-square=425.41). Other positions associated with critical incidents are mentioned in Table 5.

**Table 5:** Distribution of Study Subjects by Surgical Position The data has been represented as number (N) and percentage (%)

Position for Surgery	Number of Patients	Percentage (%)
Lateral	52	21.56
Lithotomy	100	41.49
Supine	18	7.47
Prone	70	29.05
Sitting	1	0.41

Notably, 98.75% (238) of the critical incidents were deemed preventable with existing protocols or additional resources. The most prevalent comorbidity was hypertension (59 patients, 24.48%), followed by diabetes (27 patients, 11.20%). Other comorbidities reported were chronic kidney disease (CKD) (12 patients, 4.98%), ischemic heart disease (IHD) (seven patients, 2.90%), bipolar disorder (three patients, 1.24%), nonfunctional kidney (two patients, 0.83%), aplastic anemia (one patient, 0.41%), renal calculus (one patient, 0.41%), tuberculosis (TB) (one patient, 0.41%), arteritis (one patient, 0.41%), transient ischemic attack (one patient, 0.41%), undescended testis (one patient, 0.41%), testicular abscess (one patient, 0.41%), stricture urethra (one patient, 0.41%), hepatitis B surface antigen (HbsAg) (one patient, 0.41%), and mandibulectomy (one patient, 0.41%). A total of 158 patients (65.56%) reported no comorbidities (Table 6).

**Table 6:** Distribution of Study Subjects by Comorbidities The data has been represented as numbers (N) and percentage (%)

Comorbidity	Number	Percentage (%)
Hypertension (HTN)	59	24.48
Diabetes Mellitus (DM)	27	11.20
Chronic Kidney Disease (CKD)	12	4.98
Ischemic Heart Disease (IHD)	7	2.90

Most incidents (237, 98.34%) occurred in the operating theatre. Primarily, incidents occurred during the induction phase (100 patients, 41.49%). A univariate regression analysis performed to try to correlate the critical incidence with other parameters such as ASA grade and time elapsed before the recognition of incidence did not yield significant results.

## Discussion

Among 2,541 cases examined, critical incidents were reported in 241, resulting in an incidence rate of 9.83%. Thus, the study included 241 patients undergoing various uro-surgeries in a tertiary care hospital and those who experienced a critical incident during their anesthesia. Percutaneous nephrolithotomy (PCNL) was the most common procedure performed, followed by laparoscopic nephrectomy. Over 95% of the critical incidents were deemed preventable with existing protocols or additional resources.

Despite advancements in anesthetic drugs, techniques, and equipment, anesthesia continues to pose risks of morbidity and mortality, particularly in complex surgeries such as urological procedures. Critical incident reporting and analysis in anesthesia play crucial roles in identifying risks and preventing adverse outcomes. This study assesses and analyzes critical incidents occurring during uro-surgical procedures, ultimately aiming to reduce morbidity and mortality rates. Our study predominantly involved male patients in the 31-40 age group, with a mean age of 45 years. There was however no significant association between the gender of the patient and the risk of undergoing critical incidents. The incidence rate of critical incidents in our study was 9.83%, which was higher than the 3.82% reported by Shah and Kulkarni [8] and 6.54% by Gautam and Shrestha [9]. Munting et al. (2015) found that 3.5% of anesthetic procedures resulted in critical incidents, with technical difficulties and hypotension being the most frequently documented issues [10]. In contrast, our study reported a higher incidence, particularly during the induction phase, with human factors, such as the lack of skill and vigilance, as leading causes. This variability cements the need for tailored preventive strategies in anesthetic management across different surgical contexts.

The lithotomy position was most commonly associated with incidents (41.49%), followed by the prone position (29.05%). The chi-square analysis revealed a significant association between the lithotomy position and causative factors (p<0.0001). The lithotomy position also showed the strongest association with human factors, particularly the lack of skill and judgment, suggesting that patient positioning may contribute to an increased risk of critical incidents in certain surgeries. Although this is a significant finding, it may have been due to the fact that a higher proportion of the surgeries in urology are performed in the lithotomy position and therefore may act as a confounding factor in the analysis. Most critical incidents (58.09%) occurred in ASA Grade I patients, followed by ASA Grade II (34.02%), consistent with the study by Gupta et al. (61.61%) [2]. However, Shah and Kulkarni [8] reported higher incidences in ASA Grade II patients, as did Zeng et al. [3] depicting an ascending increase from ASA 1-5, thus emphasizing that vigilance should not be relaxed for ASA 1 patients despite their perceived lower risk. Possibly, this finding may have arisen from a bias, since the highest number of patients belonged to ASA 1 [3]. Comorbidities were present in 34.44% of patients, with hypertension (24.48%) and diabetes mellitus (11.20%) being the most common. Most patients (65.56%) had no comorbidities. The presence of these comorbidities may increase perioperative risks, as Gupta et al. noted that comorbidities, more than demographic factors, may drive incident risk [2]. However, no significant associations were found in this study for comorbidities or ASA staging.

The lack of association with ASA grades, comorbidities, and specific surgery types might indicate that systemic factors or broad procedural issues are influencing incident rates, rather than patient-specific or surgery-specific factors. Regarding surgical procedures, percutaneous nephrolithotomy (PCNL) had the highest occurrence of critical incidents (28.63%), followed by laparoscopic nephrectomy (10.79%) and transurethral resection of the prostate (TURP) (9.96%). Most critical incidents (98.34%) occurred in the operating theatre, consistent with Gupta et al. (77.68%) [2] and Shah and Kulkarni (86.25%) [8], reflecting the complexity of the intraoperative period. The induction phase was the most vulnerable, with 41.49% of incidents occurring during this time, followed by 35.68% during maintenance. This aligns with the study by Agbamu et al., who identified the induction phase as having the highest frequency of critical incidents (54.8%) [11]. Notably, 54.36% of critical incidents were recognized within five minutes, proving the importance of rapid recognition and response in mitigating harm. Clinical recognition played a key role, with 74.68% of incidents identified through "clinical observation" rather than equipment monitoring, highlighting the value of anesthesiologists' clinical acumen.

The results of the chi-square test (chi-square=137.12; p<0.0001) suggest a statistically significant association between the mode of incident recognition and the time taken to recognize critical incidents. This highlights the potential for optimizing incident recognition by focusing on methods that are associated with faster detection times. The majority of incidents were classified as miscellaneous (59.75%), followed by cardiovascular (23.24%) and airway-related events (12.86%). This contrasts with Agbamu et al. [11], where cardiovascular events, particularly hypotension, were most common (41.1%), and Bajwa et al. [12], who found airway and pulmonary issues as most frequent (49%). These variations suggest that the type of critical incidents may vary based on the surgical procedure and patient population, warranting further investigation. Human factors were identified as the leading cause of incidents, with the lack of skill (43.98%), vigilance (22.41%), and judgment (16.18%) as primary contributors. These results underscore the need for ongoing education, skill development, and enhanced vigilance among anesthetists, particularly during high-risk procedures. Notably, 98.75% of incidents in this study were deemed preventable, a figure higher than a pan-European study conducted by Bielka et al., which attributed 48% of incidents as preventable [13].

Based on these findings, we recommend several strategies to advance patient safety in urological surgeries: careful monitoring during the induction phase is crucial, as this is a vulnerable period for critical incidents. The adoption of personalized anesthesia plans, tailored to consider the patient's overall health status and the specific demands of the surgery, is essential to mitigate risks. Where feasible, increasing the use of regional anesthesia can reduce complications commonly associated with general anesthesia. In addition, heightened vigilance, the presence of experienced faculty, enhanced training, and effective communication play vital roles in reducing incidents. Simulation training for potential critical incidents, along with regular mortality and morbidity meetings, can effectively lower the severity and frequency of adverse events in anesthesia practice. Lastly, the development of checklists and protocols for managing critical incidents can ensure improved patient outcomes. This study's primary strength is its focus on critical incidents during uro-surgical procedures in a tertiary care center, providing valuable insights into specific risks in this population. To our knowledge, no systematic review currently exists on critical incidents during urological procedures under anesthesia, underscoring our study's uniqueness in addressing a significant gap in the literature. However, the voluntary nature of reporting may have led to underreporting, particularly of minor incidents. The COVID-19 pandemic also disrupted elective surgeries during the study period (2019-2022), which may have affected the types and frequency of incidents observed. Data was collected both retrospectively and prospectively, which may have led to inconsistencies in the analysis, but it was minimized by maintaining standard reporting protocol for the critical incidents.

The finding that ASA 1 patients had the highest incidence of critical events contrasts with the expectation that higher ASA grades indicate greater risk and raises questions about current risk stratification methods. Future research should explore whether it is surgical complexity or intraoperative management that plays a stronger role than ASA classification alone. Additionally, clinical research is needed on how different positioning techniques and preoperative preparations could mitigate risk, as well as the further exploration of comorbidities' specific impact on perioperative safety. Across the globe, national and regional systems have been established specially for reporting anesthesia-related incidents, supplemented by efforts from international bodies such as WFSA that collect and analyze such data. These systems boost patient safety and ensure accountability among anesthesia providers. Despite these efforts, the lack of a standardized reporting protocol and a central data analytics team in many countries, particularly in LMICs, hampers the effectiveness of these systems. There is an urgent need for a unified approach to incident reporting to facilitate significant local and national improvements in care quality. Future research should focus on overcoming the barriers to developing and implementing effective reporting processes to advance global anesthesia patient safety [6]. Qualitative analyses are required to explore why certain positions are linked to higher rates of incidents and whether modifications in surgical techniques or additional safeguards can reduce these risks. The effectiveness of current safety protocols should be investigated, and whether they adequately address the risks associated with various surgical positions and stages should be questioned. Further expanding critical incident reporting to multiple centers would create more robust datasets for understanding trends and informing preventive strategies.

## Conclusions

This study underscores the necessity for implementing critical incident reporting in anesthesia as a part of quality assurance programs, as well as the need for focused training sessions for anesthesiologists aimed at enhancing patient care. Monitoring during high-risk phases, as well as the early identification of critical incidents, is crucial, and clinical observations play an important role in this. Thus, there is a rapidly growing need for enhancements in anesthesia practices, calling for plans that are meticulously tailored to meet the unique demands of surgical procedures. These findings advocate for immediate interventions to pioneer safer, more effective anesthesia protocols globally.

## References

[1] Koo CH, Ryu JH (2020). Anesthetic considerations for urologic surgeries. Korean J Anesthesiol.

[2] Gupta S, Naithani U, Brajesh SK, Pathania VS, Gupta A (2009). Critical incident reporting in anaesthesia: a prospective internal audit. Indian J Anaesth.

[3] Zeng LA, Ng SY, Thong SY (2016). Analysis of critical incidents during anesthesia in a tertiary hospital. Int J Clin Med.

[4] Mahajan RP (2010). Critical incident reporting and learning. Br J Anaesth.

[5] Battu GS (2020). NABH 5(th) edition standards - applicability to anaesthesia practices. Indian J Anaesth.

[6] Oracion EC, Endlich Y (2024). Worldwide incident reporting. Update Anaesth.

[7] (2024). IBM SPSS software. https://www.ibm.com/spss.

[8] Shah SK, Kulkarni AD (2022). Prospective analysis of intraoperative critical incidents relevant to anaesthesia in a tertiary care teaching hospital in India. J Anaesthesiol Clin Pharmacol.

[9] Gautam B, Shrestha BR (2020). Critical incidents during anesthesia and early post-anesthetic period: a descriptive cross-sectional study. JNMA J Nepal Med Assoc.

[10] Munting KE, van Zaane B, Schouten AN, van Wolfswinkel L, de Graaff JC (2015). Reporting critical incidents in a tertiary hospital: a historical cohort study of 110,310 procedures. Can J Anaesth.

[11] Agbamu PO, Menkiti ID, Ohuoba EI, Desalu I (2017). Critical incidents and near misses during anesthesia: a prospective audit. J Clin Sci.

[12] Bajwa SP, Abdullah Abdullah, Akram M, Hussain A, Safdar CA (2019). Critical anaesthetic incidents causes and analysis. Pak Armed Forces Med J.

[13] Bielka K, Kuchyn I, Frank M (2023). Critical incidents during anesthesia: prospective audit. BMC Anesthesiol.

